# A Functional *oriT* in the Ptw Plasmid of *Burkholderia cenocepacia* Can Be Recognized by the R388 Relaxase TrwC

**DOI:** 10.3389/fmolb.2016.00016

**Published:** 2016-05-03

**Authors:** Esther Fernández-González, Sawsane Bakioui, Margarida C. Gomes, David O'Callaghan, Annette C. Vergunst, Félix J. Sangari, Matxalen Llosa

**Affiliations:** ^1^Departamento de Biología Molecular, Instituto de Biomedicina y Biotecnología de Cantabria, Universidad de Cantabria, UC-SODERCAN-Consejo Superior de Investigaciones CientíficasSantander, Spain; ^2^Institut National de la Santé et de la Recherche Médicale, U1047Nimes, France; ^3^UFR de Médecine Site de Nimes, U1047, Université de MontpellierFrance

**Keywords:** bacterial conjugation, type IV secretion, *Burkholderia cenocepacia*, plasmid R388, plasmid Ptw, conjugative relaxases, origin of transfer

## Abstract

*Burkholderia cenocepacia* is both a plant pathogen and the cause of serious opportunistic infections, particularly in cystic fibrosis patients. *B. cenocepacia* K56-2 harbors a native plasmid named Ptw for its involvement in the Plant Tissue Watersoaking phenotype. Ptw has also been reported to be important for survival in human cells. Interestingly, the presence of PtwC, a homolog of the conjugative relaxase TrwC of plasmid R388, suggests a possible function for Ptw in conjugative DNA transfer. The *ptw* region includes Type IV Secretion System genes related to those of the F plasmid. However, genes in the adjacent region shared stronger homology with the R388 genes involved in conjugative DNA metabolism. This region included the putative relaxase *ptwC*, a putative coupling protein and accessory nicking protein, and a DNA segment with high number of inverted repeats and elevated AT content, suggesting a possible *oriT*. Although we were unable to detect conjugative transfer of the Ptw resident plasmid, we detected conjugal mobilization of a co-resident plasmid containing the *ptw* region homologous to R388, demonstrating the cloned *ptw* region contains an *oriT*. A similar plasmid lacking *ptwC* could not be mobilized, suggesting that the putative relaxase PtwC must act *in cis* on its *oriT*. Remarkably, we also detected mobilization of a plasmid containing the Ptw *oriT* by the R388 relaxase TrwC, yet we could not detect PtwC-mediated mobilization of an R388 *oriT*-containing plasmid. Our data unambiguously show that the Ptw plasmid harbors DNA transfer functions, and suggests the Ptw plasmid may play a dual role in horizontal DNA transfer and eukaryotic infection.

## Introduction

The *Burkholderia* genus contains more than 60 species of clinical, environmental and agro-biotechnological value (Estrada-De Los Santos et al., 2013); most of them occupy a high diversity of ecological niches, in which they act as plant pathogens and catabolically active soil saprophytes. Only two of them, *B. mallei* and *B. pseudomallei* are considered primary pathogens of animals and humans. The *B. cepacia* complex (Bcc) is a collection of currently 18 related species, sharing 97.5% rDNA sequence similarity, although only 30–60% genome-wide variability was shown by DNA-DNA hybridization (Vandamme and Dawyndt, 2011). It was initially described as one of the agents that promotes soft rot disease in onions. Importantly, Bcc bacteria pose serious health problems in vulnerable patients, particularly in individuals with cystic fibrosis (CF) or chronic granulomatous disease (CGD), and they are emerging pathogens in nosocomial infections (Coenye and Vandamme, 2003). Members of the Bcc can cause a life-threatening respiratory infection in patients with CF (Gonzalez et al., 1997; Berriatua et al., 2001; LiPuma, 2003). *B. cenocepacia* and *B. multivorans* are the most prevalent species (85–90%) of the Bcc isolated from patients with CF (LiPuma, 1998a,b; Speert, 2002). *B. cenocepacia* is correlated with increased morbidity and mortality, and it has caused several major epidemics (Goven et al., 1993).

The genome of *B. cenocepacia* strain J2315, isolated from an infected CF patient, has been sequenced (Holden et al., 2009), showing the presence of three circular chromosomes and a plasmid of 92 kb. Most of the coding DNA sequences (CDSs) present in chromosome I are involved in housekeeping functions, while chromosomes II and III [the latter recently renamed as a mega virulence plasmid (Agnoli et al., 2012)] contain CDSs with accessory functions like protective responses and horizontal gene transfer, among others (Holden et al., 2009). Many studies conducted on *B. cenocepacia* utilize another CF clinical isolate, strain K56-2 (Darling et al., 1998), which is clonally related to J2315 (Mahenthiralingam et al., 2000) but easier to grow and less resistant to antibiotics, making it more amenable to genetic manipulation. K56-2 has a shorter chromosome I due to the absence of a large duplication in J2315, and its 92 kb plasmid has one less copy of an insertion element (Varga et al., 2013).

Bacterial pathogens use secretion mechanisms for the delivery of virulence determinants (Christie and Vogel, 2000), ranging from one component systems to complex multi-component machineries. Type IV Secretion Systems (T4SS) constitute a family of molecular transporters able to deliver DNA, proteins, or nucleoprotein complexes to the extracellular milieu or other cells (prokaryotic or eukaryotic). Many T4SS mediate secretion of proteins into eukaryotic cells, being implicated in infection processes, and adaptability inside the host (Alvarez-Martinez and Christie, 2009). T4SS involved in conjugative DNA transfer (Zechner et al., 2000) are associated with a DNA transfer region (Dtr), composed by an origin of transfer (*oriT*), a coupling protein, and a conjugative relaxase; accessory nicking proteins and regulators are also often present. The relaxase catalyzes the initial and final stages of conjugative DNA transfer, cleaving the *oriT* in the donor to produce the DNA strand to be transferred, and resealing the transferred DNA in the recipient. Relaxases are defined by a series of conserved motifs, and have been classified in different families (Garcillán-Barcia et al., 2009). The *oriT* is the only DNA requirement *in cis* for mobilization of a DNA molecule. *oriTs* are usually located in intergenic regions showing a higher AT content than the rest of the molecule, to allow strand separation during initiation of single-strand transfer. *oriT* regions usually contain abundance of direct and inverted repeats (DRs and IRs), many of which have been shown to be the relaxase or accessory nicking protein binding sites (Moncalián et al., 1997; Becker and Meyer, 2000; Williams and Schildbach, 2006, 2007; Lucas et al., 2010; Wong et al., 2011). The Dtr region of plasmid R388 includes a 330 bp *oriT* and three genes transcribed from a single operon, *trwABC* (Llosa et al., 1994). The accessory nicking protein TrwA belongs to the Ribbon-helix-helix (RHH) family proteins (Moncalián and De La Cruz, 2004). TrwB is the coupling protein, interacting both with the transferred substrate and with the Type IV secretion machine (Llosa et al., 2003). The conjugative relaxase TrwC, as other members of the MOB_F_ family of relaxases, is composed by two functional domains, the N-terminus harboring the relaxase activity, and the C-terminus showing DNA helicase activity which is also required for conjugation (Llosa et al., 1996).

Two T4SS have been described in *B. cenocepacia*, the Ptw (Plant tissue watersoaking) T4SS, and the VirB/D4 T4SS (Engledow et al., 2004). The *B. cenocepacia* VirB/D4 T4SS is located on chromosome II and bears high homology with the prototypical VirB/D4 T4SS of *A. tumefaciens*. Although its function is still unknown, a possible role in DNA transfer has been reported (Zhang et al., 2009). The Ptw T4SS is encoded on a 45 kb region (Holden et al., 2009) of a native plasmid of 92 kb. Based on amino acid sequence similarity, it was proposed that the Ptw T4SS is a chimera of various translocation and/or conjugation related proteins similar to VirB/D4 and F-specific subunits (Engledow et al., 2004). One of them, named as PtwC, presents 33% amino acid identity with TrwC, the relaxase of plasmid R388 (Engledow et al., 2004). The presence of a relaxase homolog, a protein specifically associated with conjugative DNA transfer, argued for a conjugative role of the Ptw T4SS. However, to date Ptw functions have been associated with PtwC-independent secretion of a plant cytotoxic factor (Engledow et al., 2004), and intracellular survival in both professional and non-professional phagocytes (Sajjan et al., 2008).

In this study we address the characterization of the conjugative functions encoded by the 92 kb plasmid of *B. cenocepacia* K56-2 (named pK56-2 by Engledow et al., 2004), hereon referred to as the Ptw plasmid, and the possible relationship with the R388 transfer machinery. We have mapped a functional *oriT* in Ptw, suggesting that Ptw is a conjugative plasmid; therefore, Ptw may play a dual role in *B. cenocepacia*, promoting horizontal DNA transfer among bacteria and assisting infection of eukaryotic hosts.

## Materials and methods

### Bioinformatic analysis

The *B. cenocepacia* strain J2315 plasmid pBCJ2315 sequence is available in GenBank under the accession number NC_011003. The RAST (*Rapid Annotation Subsystems Technology*) program was used to re-annotate the ORFs present in the plasmid sequence (Overbeek et al., 2014). PHYRE (*Protein Homology/analogy Recognition Engine)* was used for the prediction of secondary and 3-dimensional structure of a protein amino acid sequence (Kelley et al., 2015). The phylogenetic tree was constructed using MEGA (Molecular Evolutionary Genetics Analysis) software, which employs the Maximum Likelihood method based on the JTT matrix-based model (Koichiro et al., 2011). GenScript bioinformatics software was selected to analyze AT content (http://www.genscript.com). The program Scan for Matches was used to localize IRs having at least 5 bp in length and less than two mismatches (http://www.theseed.org/servers/downloads/scan_for_matches.tgz). Other intergenic regions of the same size selected randomly from the Ptw plasmid were also analyzed for comparison.

### Bacterial strains and plasmids

Bacterial strains used in this study are listed in Table S1. All bacterial strains were grown in Lysogeny Broth (LB), supplemented with 1.5% agar for culture on solid medium, at 37°C. Selective media included the following antibiotics at the indicated concentrations: ampicillin (Ap), 100 μg/ml; chloramphenicol (Cm), 25 μg/ml (*E. coli*) or 100 μg/ml (*Burkholderia*); kanamycin (Km), 50 μg/ml; nalidixic acid (Nx), 20 μg/ml; streptomycin (Sm), 300 μg/ml; gentamicin (Gm), 10 μg/ml; and trimethoprim (Tp), 20 μg/ml (*E. coli*), or 250 μg/ml (*Burkholderia*). Plasmids are listed in Table 1. Plasmids were maintained in the *E. coli lacI*^q^ strain D1210 (Sadler et al., 1980).

**Table 1:** **Plasmids used in this work**.

**Plasmid**	**Antibiotic Resistance**	**Description**	**References**
pBBR1-MCS	Cm^R^	Broad host range vector from *Bordetella bronchiseptica*	Kovach et al., 1994
pEF022	Cm^R^	pBBR1::Ptw-*oriT*+*ptwA*+2/3*ptwB*	This work
pEF031	Cm^R^	pBBR1::Ptw-*oriT*+*ptwA*+*ptwB+ptwC*	This work
pEF033	Cm^R^	pBBR1::Ptw-*oriT* (700 bp)	This work
pEF034	Cm^R^	pBBR1::Ptw-*oriT* (700 bp)+*ptwA*	This work
pKM101	Ap^R^	R46 deletion derivative	Langer and Walker, 1981
pOX38	Km^R^	F plasmid derivative	Chandler and Galas, 1983
pSU1443	Km^R^ Tp^R^	R388::Tn5tac in *trwB*	Llosa et al., 1994
pSU1445	Km^R^ Tp^R^	R388::Tn5tac in *trwC*	Llosa et al., 1994
pSU2007	Km^R^	R388 with a Km^R^ cassette	Martinez and de la Cruz, 1988
pTn*Mob*-OCm	Cm^R^	Self-cloning minitransposon	Dennis and Zylstra, 1998

### Plasmid constructions

Plasmids were constructed using standard methodological techniques (Sambrook and Russell, 2001). *E. coli* DH5α (Grant et al., 1990) was used in all cloning procedures. Table S2 shows details of the constructions for each plasmid. All cloned DNA inserts were obtained by PCR on genomic DNA isolated from *B. cenocepacia* K56-2. Genomic DNA was extracted using Instagene Matrix (BioRad). Restriction enzymes, shrimp alkaline phosphatase, and T4 DNA ligase were purchased from Fermentas. High-fidelity Kapa Taq polymerase was purchased from KapaBiosystems. DNA sequences of all cloned PCR segments were determined (MACROGEN Inc. DNA Sequencing Service; Amsterdam, The Netherlands). Plasmid pEF031 was cloned in two sequential steps. In a first step, the 949 bp region where the possible *oriT* is located was cloned together with the hypothetical accessory nicking protein gene *ptwA* and 1269 bp of the coupling protein gene *ptwB*, adding a HindIII restriction site in *ptwB* which did not alter the predicted ORF, obtaining plasmid pEF022. In a second step, the rest of *ptwB* and *ptwC* were added to pEF022, re-creating the *ptwABC* operon.

### Colony analysis by PCR

All oligonucleotides pairs used for plasmid confirmation by PCR are summarized in Table S3. Colonies obtained from the three plasmid curing methods were checked for the presence of the Ptw plasmid by PCR, using two oligonucleotide pairs (P1-P2) and (P3-P4) amplifying *ptwC* and *pwaC* internal fragments. All the colonies tested, at least 50 for each curing method, were positive for the presence of both genes. Four oligonucleotide pairs (P5-P6; P7-P8; P9-P10; and P11-P12) were selected to detect the presence of different regions of the 45 kb *ptw* cluster in the two *B. cepacia* strains (Table S1) by PCR. K56-2 total DNA was always used as a positive control. Transconjugants obtained in the mobilization assay were checked by PCR using oligonucleotide pairs (P13-P14) to amplify the R388 *oriT* sequence of plasmid pSU1445, and (P15-P6) pair to confirm the presence of the *cat* gene in the pBBR1 plasmid.

### Plasposon insertion

For preparation of electrocompetent cells, bacteria were grown to OD_600_ = 0.5–0.6, and pelleted by centrifugation at 4°C. Two series of washes and centrifugations (6000 rpm) of 1 vol milliQ water and a final wash in 1/50 vol 10% glycerol at 4°C were applied. Cells were resuspended in 1/500 vol 10% glycerol and aliquotted in 50 μl samples. K56-2 electrocompetent cells were transformed with <10 ng of the pTn*Mob*-OCm plasposon DNA (Dennis and Zylstra, 1998) in a 0.2 cm Gene Pulser^®^ cuvette (BioRad) and subjected to an electric pulse (2.5 kV, 25 Mf, and 200 Ω) in a MicroPulser TM (BioRad). Electroporated cells were added to 1 ml LB and incubated with shaking at 37°C to allow expression of antibiotic resistance genes. After incubation cells were plated on antibiotic containing media. The pool of bacterial colonies growing on Cm plates was used as donor cells to test Ptw conjugation. Considering the size of the plasmid and the total length of the genomic DNA, it is estimated that on average one out of 100 colonies would have the plasposon inserted in the Ptw plasmid, and from those, about half would not affect any transfer-related functions. Therefore, in a mating experiment involving 10^6^ donor cells, we would expected to have at least 5 × 10^3^ cells carrying a Cm-resistant, transfer-proficient Ptw plasmid.

### Mating experiments

Standard *E. coli* quantitative mating assays were performed as described previously (Grandoso et al., 2000): equal amounts of donor and recipient strains from overnight cultures were mixed and placed on Millipore filters on a prewarmed LB agar plate for 1 h at 37°C. Strains D1210 and DH5α were used as donors and recipients, as indicated. Results are shown as the frequency of transconjugants per donor and are the mean of 3–5 independent experiments. For mating assays using *B. cenocepacia* as a donor and *E. coli* β2163 (Demarre et al., 2005) or *B. cepacia* strains as recipients, bacterial cultures were adjusted to an OD600 = 0.5, mixed at an equal ratio, washed twice and transferred to a Millipore filter on a prewarmed LB agar plate for 18 h at 37°C.

### Plant watersoaking assay

The plant watersoaking assay was used to assess the functionality of the Ptw T4SS in *B. cenocepacia*, as described (Engledow et al., 2004). Several onion types, like echalote, red onion and white onion were used throughout the study, and no significant differences were found among them, so all experiments were performed with white onions. Bacterial suspensions of *B. cenocepacia* strains were adjusted to OD_600_ = 0.5 and individual onion scales were wounded on the abaxial (inner) surface with a sterile blade. 10 μl of bacterial suspension (10^6^ c.f.u per scale) were inoculated into the wound. Sterile double-distilled water was used as a negative control. Onion scales were placed on a sheet of sterile aluminum foil in containers containing Whatman paper towels moistened with sterile distilled water, sealed, and incubated at 37°C. Ptw activity was estimated by the appearance of water drops on the onion tissue at 24 hpi.

### Plasmid curing

In order to cure the Ptw plasmid from *B. cenocepacia*, bacteria were incubated under different stress conditions, where mutations in essential plasmid genes can be induced, promoting the loss of the plasmid. Two methods using high temperatures and a third one growing bacteria in the presence of the mutagenic agent ethidium bromide were used: *(i) Growth on TN Medium* (Gonzalez et al., 1997). Bacteria containing the plasmid to be cured were grown in TN broth [5 g Tryptone, 1 g Dextrose (D-Glucose), 2.5 g Yeast extract, and 8.5 g NaCl per litter] for 18 h at 37°C with shaking (200 r.p.m). Bacteria were subcultured into pre-warmed TN broth (42–44°C) to a final concentration of 10^4^ c.f.u ml^−1^ and grown with shaking in a water bath (at 42–44°C) for 18 h. Temperature-treated cultures were diluted and plate on TN agar. Individual colonies were checked by PCR. *(ii) Growth at High Temperature* (Asheshov, 1966). Bacteria were grown overnight at 37°C. Overnight cultures were diluted ¼ in fresh LB medium for 1 ½ h. Samples of this culture were added to tubes containing 5 ml of fresh LB medium previously pre-warmed at 37°C and at 43–44°C respectively. Tubes were then incubated at the appropriate temperature for 5 ½ h. Cultures were then diluted and spread on LB agar plates and incubated at 37°C overnight; the resulting colonies were then tested for the loss of the native plasmid by PCR. *(iii) Growth with Ethidium Bromide* (Crameri et al., 1986). Ethidium bromide (25 μg/ml) was added to a *B. cenocepacia* liquid culture and incubated with shaking at 37°C overnight protected from the light. A 1/10 dilution of the overnight culture was prepared with fresh LB medium supplemented with Ethidium bromide. This procedure was repeated over 15 days; every 2 days, dilutions of the culture were plated, and colonies were checked for the presence of the plasmid by PCR.

## Results

### Bioinformatic analysis of the Ptw plasmid

A preliminary annotation of the Ptw plasmid sequence for *B. cenocepacia* J2315 was available (Holden et al., 2009). We have used the RAST Bioinformatic Program to obtain a detailed annotation of the ORFs present in the plasmid, based on a comparative study against the NCBI database. As previously reported (Engledow et al., 2004; Holden et al., 2009), a 45 kb region was found to encode genes with similarity to the F plasmid T4SS genes (Lawley et al., 2003). However, the adjacent DNA region to T4SS genes, which included the putative relaxase and coupling protein genes previously described, *ptwC* and *ptwD4*, showed higher similarity to the Dtr region of conjugative plasmid R388: PtwC shows 33% of amino acid identity with R388 relaxase TrwC, and 27% with relaxase TraI of the F plasmid. PtwD4 presents 20% identity with VirD4 from *A. tumefaciens*, 28% with TraD of the F plasmid, and 30% with R388 TrwB. Due to the stronger similarity to TrwB, PtwD4 will be named PtwB from here on.

**Figure 1:**
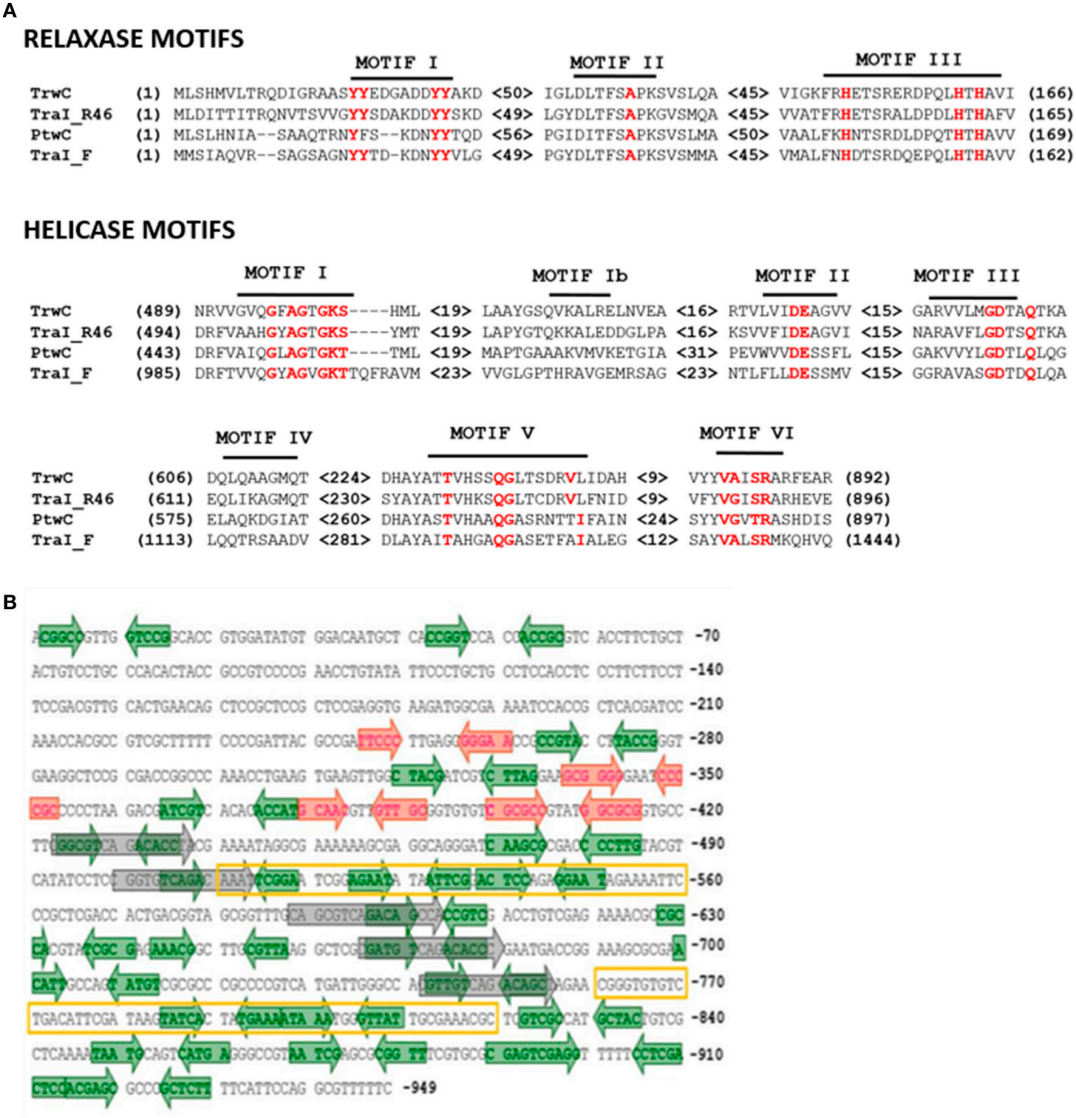
**Conserved motifs in PtwC and related proteins and features of the hypothetical *oriT* region. (A)** Conserved motifs in the relaxase and DNA helicase domains, with the most conserved residues highlighted in red. **(B)** Features in hypothetical Ptw *oriT* region (coordinates 66.438–65.490 in Accession number NC_011003.1). IRs with one mismatch are represented in green. A set of DRs of 15 bp with three mismatches are represented in gray. High AT regions are marked with a yellow box.

Both R388-TrwC and F-TraI belong to the MOB_F_ family of relaxases, defined by three common motifs based on the known atomic structure of the TrwC relaxase and similar proteins (Francia et al., 2004). CLUSTALW alignment of PtwC and its homologs showed that the three relaxase motifs were conserved in PtwC (Figure 1A). The homology between TrwC and PtwC in this amino terminal domain goes up to 42%, while the C-terminal domains share 29% amino acid identity. In addition, PtwC shares the seven conserved amino acid motifs in the C-terminal domain which define the DNA helicase superfamily I (Matson, 1990; Figure 1A). The MEGA5 program (Koichiro et al., 2011) was used to determine its phylogenetic position in MOB_F_ family (Figure S1), confirming that PtwC belongs to the MOB_F_ family, and R388-TrwC is its closest relative out of the *Burkholderia* spp homologs.

In most conjugative systems, the *oriT* is located close to the relaxase; accessory nicking proteins are also often encoded in this DNA region. Analysis of the DNA region upstream of *ptwB* led to the identification of a small ORF of 429 bp (pBCA060) coding for a hypothetical protein with a predicted RHH structure related to other members of the RHH superfamily (Heidelberg et al., 2000), and thus a likely accessory nicking protein of PtwC; we named it PtwA after TrwA, the accessory nicking protein of TrwC, although both proteins do not share significant amino acid identity. The most likely region to contain the Ptw *oriT* sequence would be in a 949 bp intergenic region located upstream of *ptwA*. It contains DNA segments with high AT content (squared regions in Figure 1B), candidates to contain the *nic* site, and two times more IRs than in a randomly selected intergenic region of the same size (not shown), especially in the region including the two AT-rich DNA segments (Figure 1B). All these features lead us to propose this as a candidate region to contain the Ptw *oriT*.

**Figure 2:**
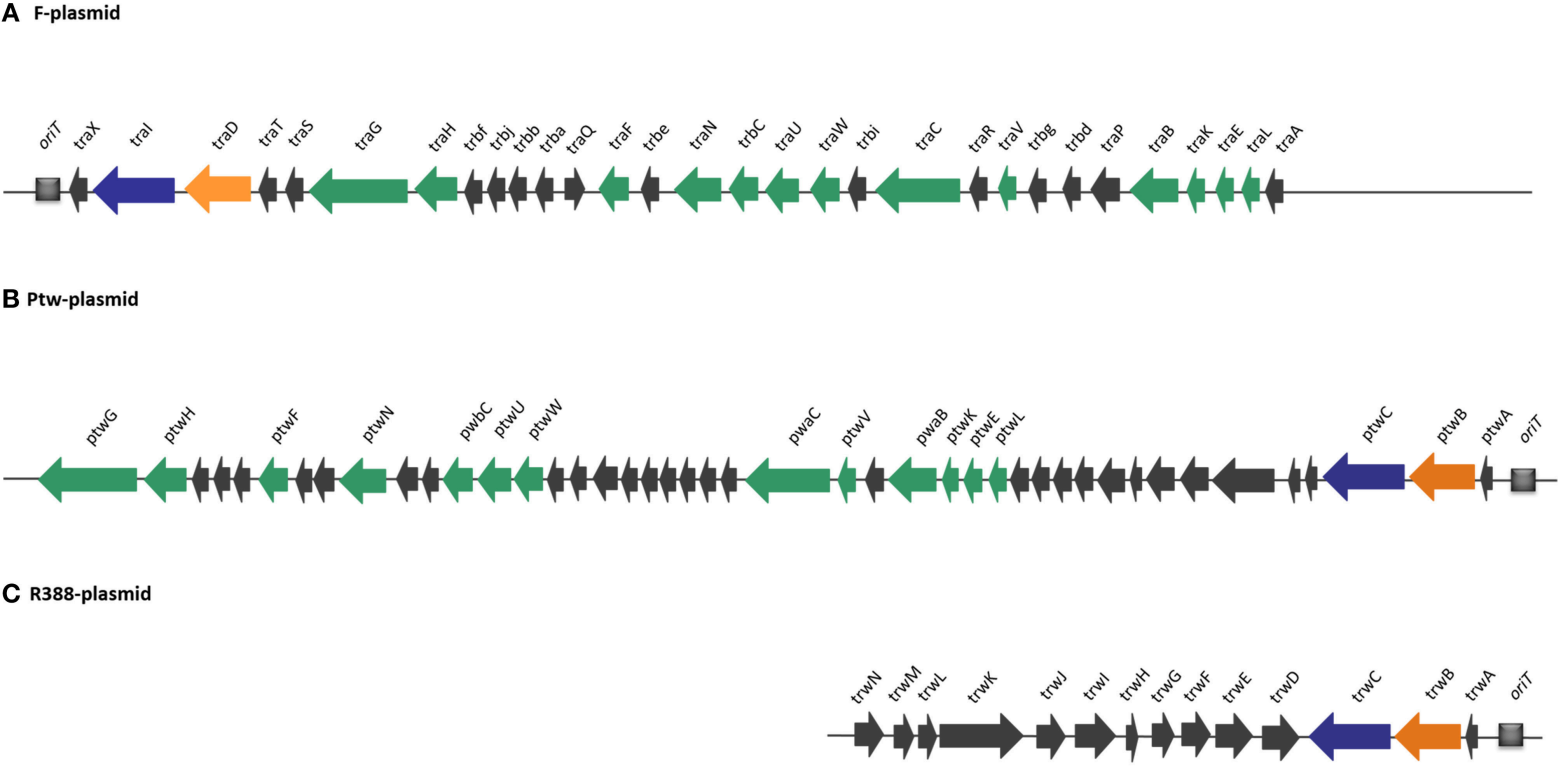
**Comparison of DNA transfer regions of conjugative plasmids (A) F, (B) Ptw, and (C) R388, highlighting structural and sequence homologies**. Homologous ORFs are highlighted in a color-coded format: T4SS genes are shown in green, relaxases in blue, and coupling proteins in orange. Ptw genes are named based on their closest homologs in F and R388 plasmids.

Compiling the results of the bioinformatics analysis, we propose that the Ptw plasmid codes for a conjugative transfer region with a T4SS related to that of the F plasmid, while the Dtr region is closely related to that of the R388 transfer system (Figure 2).

### Involvement of the Ptw plasmid in conjugative DNA transfer

Our next goal was to experimentally test conjugative transfer of the Ptw plasmid. Entry exclusion is a common phenomenon in conjugative DNA transfer, which prevents conjugation into a recipient cell already containing the conjugative plasmid present in the donor (Garcillán-Barcia and de la Cruz, 2008). To prevent this possible effect, a Ptw-free recipient strain was required. We attempted to cure a *B. cenocepacia* strain of the plasmid by three different methods (see Materials and Methods); colonies were checked by PCR and assayed for ability to induce the plant watersoaking phenotype; not a single colony out of about 150 screened colonies (50 from each method) had lost the plasmid.

The Ptw plasmid has been described only in *B. cenocepacia* (Engledow et al., 2004). Therefore, other bacterial species were selected to be used as recipients in the conjugation assays (Table S1). No Ptw homologs are present in the five available *B. cepacia* genomes, so we used two ATCC *B. cepacia* strains as recipients. Since their genomic sequence is not available, we checked for the presence of identical or highly homologous *ptw* sequences by PCR amplification of four different regions of the 45 kb *ptw* cluster, which were all negative. To detect Ptw plasmid transfer, we inserted a pTn*Mob*-Cm plasposon by electroporation in K56-2. Colonies resistant to Cm were used as donors in conjugation to K56-2, *B. cepacia* 322 and *E. coli* β2133. After a series of transfer experiments using about 5 × 10^9^ Cm-resistant donor colonies, transconjugants were never obtained. The absence of Ptw DNA transfer could be due to natural repression or lack of induction of this transfer system.

It cannot be discarded that transconjugants were not detected in *B. cepacia* due to lack of replication of the Ptw plasmid, or incompatibility with some resident plasmid. These limitations would be overcome by using a different mobilizable replicon. The putative Ptw Dtr region, consisting of *ptwABC* and *oriT*, was cloned on the broad-host-range pBBR1 replicon (Kovach et al., 1994), and a plasmid carrying the Ptw *oriT* but without the putative relaxase *ptwC* was also constructed. The plasmids were introduced in *B. cenocepacia* K56-2, which naturally contains the Ptw plasmid, providing the *ptw* transfer genes also *in trans*. Recipient strains harboring a trimethoprim resistant plasmid (pSU1445; Table 1) were used to allow selection of transconjugants. Mating assays were carried out as described in Experimental Procedures, and results are shown in Table 2. When assaying mobilization of a plasmid carrying the putative Ptw *oriT, ptwA*, and a truncated copy of *ptwB*, no transconjugants were ever obtained, in spite of the presence of the Ptw plasmid which provides the complete putative transfer machinery. However, a pBBR-plasmid containing the complete Ptw Dtr region was mobilized to both *B. cepacia* strains; no mobilization to *B. cenocepacia* K56-2 or to *E. coli* was detected. Transconjugants were verified by PCR amplification to confirm the presence of both plasmid pSU1445 and the mobilized plasmid. This result proves the presence of a functional *oriT* in the Ptw plasmid.

**Table 2:** **Mating assays to test mobilization of the Ptw Dtr region**.

**Mobilizable plasmid**	**Recipient strain**			
	***B. cenocepacia* K56-2 (pSU1445)**	***B. cepacia* 322 (pSU1445)**	***B. cepacia* 4461 (pSU1445)**	***E. coli* β2163 (pSU1445)**
pEF022 (pBBR1:*Ptw oriT*+ *ptwA +2/3 ptwB*)	<2.8 × 10^−8^	<1.9 × 10^−8^	<2.4 × 0^−8^	<3.1 × 10^−8^
pEF031 (pBBR1:*Ptw oriT* + *ptwA*BC)	<2.2 × 10^−8^	**4.4 × 10^−5^**	**3.1 × 10^−5^**	<2.7 × 10^−8^

*B. cenocepacia K56-2 was used as donor strain. Conjugation frequencies (transconjugants/donor) represent the mean of at least three independent experiments. Positive mobilization frequencies are highlighted in bold*.

### Recognition of the Ptw *oriT* by the conjugative relaxase TrwC

It has previously been reported that conjugative systems can use heterologous T4SS with different affinities: the TrwC-DNA complex could be secreted through the T4SS of different conjugative plasmids, and moreover, it could also use the T4SS of *Bartonella henselae* to be transferred to human cells (Llosa et al., 2003; Fernández-González et al., 2011). Mobilization of a plasmid coding for the Ptw Dtr components by other conjugative T4SS was therefore tested. Plasmids containing different parts of the Ptw Dtr were tested for mobilization between *E. coli* strains. Three different conjugative plasmids were used as helper plasmids in the donor strain: pSU2007 (R388 derivate) (Martinez and de la Cruz, 1988), pOX38-Km (F derivative) (Chandler and Galas, 1983), and pKM101 (R46 derivative; Langer and Walker, 1981; Table 1), all coding for relaxases sharing homology with PtwC (Figure 1). Results are shown in Table 3. While no transconjugants were observed with the F or R46 transfer systems, a low level of mobilization of the Ptw-*oriT* containing plasmids was observed when the R388 transfer system was present in the donor cell. Transconjugants were analyzed by restriction analysis (data not shown), showing both the mobilizable and helper plasmids as separate entities in the transconjugants (clearly distinguishable by their different copy number), thus ruling out plasmid conduction by cointegrate formation with the conjugative plasmid. Restriction analysis with HindIII and SacI rendered the expected fragments for the helper pSU2007 (32.5 kb) and mobilizable plasmids pEF022 or pEF031: 2640 bp fragment in all cases (*ptw oriT* + *ptwA* + *2/3 ptwB*) plus a fragment of 4700 or 8800 bp, respectively.

**Table 3:** **Mobilization of plasmids carrying the Ptw-*oriT* by different conjugative plasmids between *E. coli* strains**.

**Plasmids in donor**				**Transfer frequency**	
**Helper** ***(TRA Rel CP)***^**a**^		**Mobilizable** ***(Ptw Dtr)***		**Helper**	**Mobilizable**
pKM101	IncN TraI TraD			0.21 ± 0.11	
pKM101	IncN TraI TraD	pBBR1		0.19 ± 0.12	<3.9 × 10^−8^
pKM101	IncN TraI TraD	pEF022	*Ptw*-*oriT*+*ptwA*^b^	0.19 ± 0.11	<2.3 × 10^−8^
pKM101	IncN TraI TraD	pEF031	*Ptw*-*oriT*+*ptwABC*	0.25 ± 0.08	<2.5 × 10^−8^
pOX38	IncF TraI TraD			0.51 ± 0.03	
pOX38	IncF TraI TraD	pBBR1		0.34 ± 0.07	<5.6 × 10^−8^
pOX38	IncF TraI TraD	pEF022	*Ptw*-*oriT*+*ptwA*^b^	0.36 ± 0.37	<2.2 × 10^−8^
pOX38	IncF TraI TraD	pEF031	*Ptw*-*oriT*+*ptwABC*	0.19 ± 0.02	<1.9 × 10^−8^
pSU2007	IncW TrwC TrwB			0.11 ± 0.08	
pSU2007	IncW TrwC TrwB	pBBR1		0.20 ± 0.16	<1.3 × 10^−8^
pSU2007	IncW TrwC TrwB	pEF022	*Ptw*-*oriT*+*ptwA*^b^	0.12 ± 0.11	**1.5 × 10^−7^± 1.1 × 10^−7^**
pSU2007	IncW TrwC TrwB	pEF031	*Ptw*-*oriT*+*ptwABC*	0.19 ± 0.11	**8.2 × 10^−8^± 5.0 × 10^−9^**
pSU2007	IncW TrwC TrwB	pEF033	*Ptw*-*oriT*(700)	0.21 ± 0.06	**7.9 × 10^−8^± 1.3 × 10^−8^**
pSU2007	IncW TrwC TrwB	pEF034	*Ptw*-*oriT*(700) +*ptwA*	0.16 ± 0.15	**1.3 × 10^−7^± 1.0 × 10^−7^**
pSU1443	IncW TrwC			<1.5 × 10^−7^	
pSU1443	IncW TrwC	pBBR1		<2.2 × 10^−7^	<3.1 × 10^−8^
pSU1443	IncW TrwC	pEF031	*Ptw*-*oriT*+*ptwABC*	<2.9 × 10^−7^	<4.5 × 10^−8^
pSU1445	IncW TrwB			<2.8 × 10^−7^	
pSU1445	IncW TrwB	pBBR1		<2.5 × 10^−7^	<2.8 × 10^−8^
pSU1445	IncW TrwB	pEF022	*Ptw*-*oriT*+*ptwA*^b^	<2.9 × 10^−7^	<3.6 × 10^−8^
pSU1445	IncW TrwB	pEF031	Ptw-*oriT*+*ptwABC*	<3.1 × 10^−7^	<1.9 × 10^−8^

*DH5α was used as a donor and D1210 as a recipient. Transfer frequencies (transconjugants/donor) represent the mean ± SD of three independent experiments*.

a *Description of the Helper plasmid: TRA transfer system (defined by plasmid Inc group), Rel relaxase, CP coupling protein*.

b *A truncated ptwB copy was also present, which was omitted for clarity. Positive mobilization frequencies are highlighted in bold*.

Surprisingly, similar results were obtained when the mobilizable plasmid carried only the Ptw *oriT, ptwA*, and 2/3 of *ptwB*, instead of the complete Ptw Dtr region (Table 3, compare mobilization by pSU2007 of pEF022 and pEF031). Thus, mobilization through the T4SS of R388 is not due to PtwC, but presumably to recognition of the Ptw *oriT* by TrwC *in trans*. To confirm if mobilization is TrwC dependent, a TrwC-deficient R388 plasmid (pSU1445) was used as helper; in this case, no plasmid mobilization was detected (Table 3). These assays also show that there is no functional complementation of TrwB or TrwC by PtwB and PtwC, at least *in trans*, since R388 *trwB*- (pSU1443) and R388 *trwC*- (pSU1445) deficient plasmids cannot be mobilized in the presence of a plasmid harboring the Ptw Dtr region (Table 3, negative transfer frequency of pSU1443 and pSU1445 in the presence of pEF031).

Mobilization of Ptw-*oriT* containing plasmids by R388 allowed us to refine mapping of the Ptw *oriT*. Plasmids were constructed carrying only the region where the *oriT*-like features were found, with and without *ptwA*. A plasmid carrying region 65.490–66.190 of the Ptw-*oriT* (nucleotides 249–949 in Figure 1B) was mobilized by TrwC as efficiently as the plasmid containing the complete Ptw Dtr region (Table 3, compare mobilization frequency of pEF031 and pEF033 by pSU2007).

## Discussion

Previous studies proposed that the T4SS encoded on the Ptw plasmid of *B. cenocepacia* played a role in interactions with the eukaryotic host. The T4SS was associated with the plant tissue watersoaking phenotype, produced by unknown translocated effector proteins (Engledow et al., 2004). In addition, the Ptw T4SS was shown to have a role in intracellular survival and replication of *B. cenocepacia* inside macrophages (Sajjan et al., 2008), although Valvano and colleagues were unable to show defects in intracellular survival in *B. cenocepacia* mutants lacking all possible secretion systems (Valvano, 2015). However, the presence of the *ptwC* gene coding for a putative conjugative relaxase, not involved in the Ptw phenotype (Engledow et al., 2004), led us to hypothesize that Ptw is a conjugative plasmid. Relaxases are the signature proteins of conjugative systems; all known close homologs are involved in horizontal DNA transfer (Garcillán-Barcia et al., 2009). PtwC belongs to the MOB_F_ family (Garcillán-Barcia et al., 2009) of relaxases-helicases, according to phylogenetic analysis (Figure S1). Conservation in PtwC of the signature motifs associated to relaxase and DNA helicase activity (Figure 1) reinforce the idea that PtwC is an active conjugative relaxase.

A straightforward way to determine the role of the Ptw plasmid would be to cure it from *B. cepacia* strains; however our repeated attempts were unsuccessful. Low-copy-number plasmids have developed a number of mechanisms to promote their stable maintenance, such as Toxin-Antitoxin (TA) systems (Yamaguchi et al., 2011). The Ptw plasmid indeed encodes genes showing similarity to the toxin and antitoxin of the VapBC TA system of *Mycobacterium tuberculosis* (Ramage et al., 2009), which could explain the failure to cure the Ptw plasmid. Similarly, recent studies have shown that the pC3 mega-plasmid (previously chromosome 3) in *B. cenocepacia* K56-2 showed unexpected stability due to the presence of a toxin-antitoxin system, which made it difficult to cure this non-essential plasmid from K56-2, in contrast to other Bcc strains (Agnoli et al., 2012).

The Ptw T4SS was reported to be a chimera between VirB and F-like specific subunits (Engledow et al., 2004). Our bioinformatic analyses show that the plasmid possesses all the components of a conjugative system, composed of a T4SS gene set related to that of the F plasmid, and a Dtr region whose closest homolog is that of plasmid R388 (Figure 2). In the Dtr region, upstream the putative coupling protein and relaxase previously reported (Engledow et al., 2004), we described a protein, PtwA, with similar size to accessory nicking proteins, predicted to have the characteristic Ribbon-Helix-Helix secondary structure. More importantly, we delimited a region upstream of this putative accessory nicking protein candidate to be the Ptw *oriT*, due to its high AT content and the elevated number of IRs and DRs (Figure 1).

Despite the presence of a DNA transfer region, Ptw plasmid conjugation could not be detected under different test conditions. However, we detected mobilization of a Ptw-Dtr containing plasmid introduced in the host of the Ptw plasmid. It is reasonable to assume that mobilization will occur through the Ptw T4SS, although we cannot discard that the plasmid may be using the VirB T4SS, previously shown to be involved in DNA transfer (Zhang et al., 2009). Whatever the T4SS used for secretion, the absence of mobilization of the Ptw plasmid, which includes the Dtr region, suggests that self-transfer is repressed *in cis*. This is also the case for one of the symbiotic plasmids (pSym) present in *Rhizobium etli*, which possesses a full set of genes involved in conjugation. Plasmid transfer has never been detected under laboratory conditions, although there are several lines of evidence for the movement of pSym plasmids among naturally occurring rhizobial populations (Wernegreen and Riley, 1999). The absence of conjugation in this symbiotic plasmid has been explained by the presence of a conjugative repressor, *rctA*, which inhibits the conjugation of the symbiotic plasmid by decreasing *virB* transcription (Sepúlveda et al., 2008). No similar genes to *rctA* have been described until now in the Ptw plasmid, however the presence of such a repressor cannot be discarded.

Mobilization of the plasmid containing the Ptw Dtr occurred to two different *B. cepacia* strains, but not to *B*. *cenocepacia* or to *E*. *coli* (Table 2). The absence of transfer to *B*. *cenocepacia* could reflect an entry-exclusion phenomenon, frequent in bacterial conjugative plasmids (Garcillán-Barcia and de la Cruz, 2008), due to the presence of the Ptw plasmid in the recipient. The absence of transfer to *E. coli* could reflect a narrow host range of this conjugative system, similar to that of the F plasmid to which the T4SS is most closely related (Zhong et al., 2005).

Plasmids containing the putative *oriT* but no *ptwC* were not mobilized (Table 2), supporting the sequence homology and phylogenetic analysis which indicate that PtwC is a conjugative relaxase. Since, *ptwC* is present in the co-resident Ptw plasmid and no Ptw transfer is detected, PtwC may act only *in cis*, as previously reported for other conjugative relaxases (Pérez-Mendoza et al., 2006; Cho and Winans, 2007). Alternatively, this could be due to *cis*-repression of the *ptwC* copy present in the Ptw plasmid, as discussed above. The *ptwC* copy present in the multicopy plasmid would escape repression *in trans*, or if the presence of multiple *ptwC* copies outnumbers the repressor, leading to partial expression (in accordance with the low transfer frequencies obtained).

Interestingly, we showed that the Ptw *oriT* can be also recognized by the conjugative relaxase of plasmid R388, TrwC (Table 3), while the reverse is not true: PtwC does not act on R388 *oriT*, at least *in trans*. The fact that TrwC can mobilize Ptw-*oriT* containing plasmids *in trans* while PtwC cannot, can be explained by the different nature of both relaxases. *cis*- or *trans*-acting is an attribute of the relaxase, not the *oriT*. PtwC may act *in cis* as has been reported for a few other relaxases, but most of them act *in trans*, including TrwC (Llosa et al., 1996). If alternatively the lack of trans-mobilization by PtwC were due to *cis*-repression of the *ptwC* gene, as suggested above, this repression would obviously not affect the *trwC* copy present in a different plasmid.

Traditionally considered as highly sequence specific, conjugative relaxases have been shown to act on related sequences with relaxed specificity (Jandle and Meyer, 2006; Fernández-López et al., 2013; O'Brien et al., 2015; Pollet et al., 2016). However, it is surprising that TrwC can act on another *oriT* with no apparent homology to its natural target; we have not found any sequence within the 700 bp defined as the Ptw *oriT* which resembles the R388 *nic* site and the surrounding 17 bp which are required for TrwC binding and nicking (Lucas et al., 2010). TrwC has been reported to recognize sequences which differ from its natural target (Agúndez et al., 2012), and it is also efficiently recruited by heterologous T4SS (Llosa et al., 2003; Fernández-González et al., 2011), so this result adds to previous ones to highlight the promiscuity of this enzyme and its conjugative transfer system. In addition, this result opens up the possibility that the Ptw plasmid can be transferred in nature by the conjugative system of a horizontally transmitted promiscuous plasmid such as is R388.

Earlier reports have suggested a role for the Ptw T4SS in the Plant Tissue Water soaking phenotype and survival in mammalian host cells through the secretion of putative effector proteins (Sajjan et al., 2008). Our results unambiguously show that the Ptw plasmid encodes an active *oriT* adjacent to the *ptw* T4SS gene cluster. It would be interesting to find out whether conjugative transfer during infection of mammalian hosts would contribute to disease. A role for the Ptw plasmid in transfer of DNA and effector proteins may give an advantage to *B. cenocepacia* in the environment, while at the same time contributing to their opportunistic character.

## Author contributions

EF contributed to the experimental set-up, analysis, interpretation of data and drafting the work; SB and MG contributed to the experimental results; DO and FS contributed to the conception of the work and critical revision of the manuscript; FS, AV, and ML contributed to the design of the work, interpretation of data, drafting, and revising the manuscript. All the authors approved the final version of the manuscript.

## Funding

This work was supported by grants BIO2013-46414-P from the Spanish Ministry of Economy and Competitiveness to ML and BFU2011-25658 from the Spanish Ministry of Science and Innovation to FS. U1047 is supported by INSERM and the Université de Montpellier. AV was recipient of a “Chercheur d'avenir” award from La Region Languedoc-Roussillon, and SB was supported by a doctoral fellowship co-funded by the Region Languedoc-Roussillon and INSERM.

### Conflict of interest statement

The authors declare that the research was conducted in the absence of any commercial or financial relationships that could be construed as a potential conflict of interest.

## Abbreviations

Ptw: plant tissue watersoaking
Dtr: DNA transfer and replication
*oriT*: origin of transfer
T4SS: Type IV Secretion System.
