# Supplementary material for: A Functional oriT in the Ptw Plasmid of Burkholderia cenocepacia Can Be Recognized by the R388 Relaxase TrwC
Source: Front Mol Biosci. 2016 May 3;3:16. doi: 10.3389/fmolb.2016.00016 (PMC4853378; doi:10.3389/fmolb.2016.00016)
Supplement: Supplementary file 1 [file DataSheet1.docx]

Supplementary Material

**A functional *oriT* in the Ptw plasmid of *Burkholderia cenocepacia* can be recognized by the R388 relaxase TrwC**

Esther Fernández-González^1^, Sawsane Bakioui^2,3^, Margarida Gomes^2,3^, David O´Callaghan^2,3^, Annette C. Vergunst*^2,3^, Félix J. Sangari^1^_,_ and Matxalen Llosa*^1^

***Correspondence:**

Matxalen Llosa: [llosam@unican.es](mailto:llosam@unican.es)

Annette Vergunst: [annette.vergunst@umontpellier.fr](mailto:annette.vergunst@umontpellier.fr)

# Supplementary Figures and Tables

## Supplementary Tables

**Table S1.** Bacterial strains.

| **Strain** | **Genotype** | **Reference** |
| --- | --- | --- |
| *Burkholderia cepacia* CECT 322 | *Burkholderia cepacia* isolated from *ex* forest soil, Seven Mile Stretch, Trinidad. | CECT 322 |
| *Burkholderia cepacia* CECT 4461 | *Burkholderia cepacia* isolated from *ex* soil. | CECT 4461 |
| *Burkholderia cenocepacia* K56-2 | ET12, Toronto, Canada, CF | (Darling et al., 1998) |
| *Escherichia coli* D1210 | Sm^R^*; recA hspR hsdM rpsl lacI^q^* | (Sadler et al., 1980) |
| *Escherichia coli* DH5α | Nx^R^*; F^-^ endA1 hsdR17 supE44 thi-1 recA1 gyrA96 relA1 Δ(argF-lacZYA) U 169 Φ80dlac ΔM15* | (Grant et al., 1990) |
| *Escherichia coli β2163* | (F−) RP4-2-Tc::Mu_*dapA*::(*erm-pir*) (Km^R^ Em^R^) | (Demarre et al., 2005) |

**Table S2.** Plasmids constructed for this work.

|  | |  | **Construction ^(1)^** | | |
| --- | --- | --- | --- | --- | --- |
| **Plasmid** | | **Description** | **Vector** | | **Digestion/oligonucleotides** |
| pEF022 | pBBR1::Ptw-*oriT*+*ptwA*+2/3*ptwB* | | pBBR1 | SacI-HindIII  CCAGAGCTCCCGTGGATATGTGGACAATGC  CCACAAGCTTCTGCAGGGTCGG | |
| pEF031 | | pBBR1::Ptw-*oriT*+*ptwA*+*ptwB+ptwC* | pEF022 | | HindIII-ClaI  CCACAAGCTTGATATCATGAAGCTCGCG  CCAATCGATTCAAATTTCATGCTGTTCGCGCT |
| pEF033 | | pBBR1::Ptw-*oriT* (700bp) | pBBR1 | | SacI-HindIII  CCAGAGCTCCGATTCCCTTGAGCGGGA  CAAAAGCTTGAAAAACGCCTGGAATGAAAG |
| pEF034 | | pBBR1::Ptw-*oriT* (700bp) + *ptwA* | pBBR1 | | SacI-HindIII  CCAGAGCTCCGATTCCCTTGAGCGGGA  CAAAGCTTTCATTTCTGGCTACCCCGCA |

| **(1)** The first column lists the vector plasmids; the second column indicates the restriction enzymes used for cloning and the oligonucleotides used for PCR amplification of the desired fragment, with the restriction sites underlined. The DNA source for the inserts was in all cases genomic DNA from *B. cenocepacia* K56-2. |
| --- |

**Table S3.** Oligonucleotides used for PCR confirmation of the presence of plasmids.

| **Primer** | **Amplified region in** | **Sequence** |
| --- | --- | --- |
| P1 | ptwC | 5´ TACGGCGTATATGACCGACA 3´ |
| P2 |  | 5´ CGTTGCGCTTCAGTTCAATA 3´ |
| P3 | pwaC | 5´ ATGAATTCCGGCATGAAGAC 3´ |
| P4 |  | 5´ GAGTTCTGGGCATCTTCGAG 3´ |
| P5 | *oriT + ptwA* | 5´ CCAGAGCTCCCGTGGATATGTGGACAATGC 3´ |
| P6 |  | 5´ CCACAAGCTTCTGCAGGGTCGG 3´ |
| P7 | *ptwC* | 5´ CCCGCCGTGAGCGCATTCGC 3´ |
| P8 |  | 5´ GGGTCCTGCCGAACGGTCTC 3´ |
| P9 | *ptwB + ptwC* | 5´ CCACAAGCTTGATATCATGAAGCTCGCG 3´ |
| P10 |  | 5´ CCAATCGATTCAAATTTCATGCTGTTCGCGCT 3´ |
| P11 | *PtwC internal fragment* | 5’ GGTGCCGCTGCCGGATGAGAA 3’ |
| P12 |  | 5’ TCCACCCCCTGCAGCTGAAGC 3’ |
| P13 | *R388 oriT* | 5´ CCAAGTCGACCTCTCCCGTAGTGTTACT 3´ |
| P14 |  | 5´ CCAAAGTCTACTCATTTCTGCATCATTGT 3´ |
| P15 | *cat pBBR1* | 5´ GGGAAACCTGTCGTGCCAGCTGCATTAATG  AATCGGCCAAGGCACCAATAACTGCCT 3´ |
| P16 |  | 5´ TTACGCCGTGGGTCGATGTTTGATGTTATGGAGC  AGCAACGATGGAGAAAAAAATCACTGGATA 3´ |

## Supplementary Figure

**Legend to Figure S1. Phylogenetic analysis of PtwC and related proteins.** Phylogenetic tree determined by Molecular Phylogenetic analysis by Maximum Likelihood method. The tree was obtained by alignment of the relaxase domains of relaxases of the families MOB_Q_, MOB_P_ and MOB_F_ (Garcillán-Barcia et al., 2009), as well as other PtwC or TrwC proteins present in *Burkholderia* spp. The tree is drawn to scale, with branch lengths measured in the number of substitutions per site. MOB F, Q and P refer to the MOB family of relaxases, as described by (Garcillán-Barcia et al., 2009).

**References:**

Darling, P., Chan, M., Cox, A. D., and Sokol, P. A. (1998). Siderophore production by cystic fibrosis isolates of *Burkholderia cepacia*. *Infect. Immun.* 66, 874–877.

Demarre, G., Guérout, A. M., Matsumoto-Mashimo, C., Rowe-Magnus, D. A., Marlière, P., and Mazel, D. (2005). A new family of mobilizable suicide plasmids based on broad host range R388 plasmid (IncW) and RP4 plasmid (IncPα) conjugative machineries and their cognate *Escherichia coli* host strains. *Res. Microbiol.* 156, 245–255. doi:10.1016/j.resmic.2004.09.007.

Garcillán-Barcia, M. P., Francia, M. V., and De La Cruz, F. (2009). The diversity of conjugative relaxases and its application in plasmid classification. *FEMS Microbiol. Rev.* 33, 657–687. doi:10.1111/j.1574-6976.2009.00168.x.

Grant, S. G., Jessee, J., Bloom, F. R., and Hanahan, D. (1990). Differential plasmid rescue from transgenic mouse DNAs into *Escherichia coli* methylation-restriction mutants. *Proc. Natl. Acad. Sci. U. S. A.* 87, 4645–4649. doi:10.1073/pnas.87.12.4645.

Sadler, J. R., Tecklenburg, M., and Betz, J. L. (1980). Plasmids containing many tandem copies of a synthetic lactose operator. *Gene* 8, 279–300. doi:10.1016/0378-1119(80)90005-0.
