# Supplementary figures and images for: A Functional oriT in the Ptw Plasmid of Burkholderia cenocepacia Can Be Recognized by the R388 Relaxase TrwC
Source: Front Mol Biosci. 2016 May 3;3:16. doi: 10.3389/fmolb.2016.00016 (PMC4853378; doi:10.3389/fmolb.2016.00016)

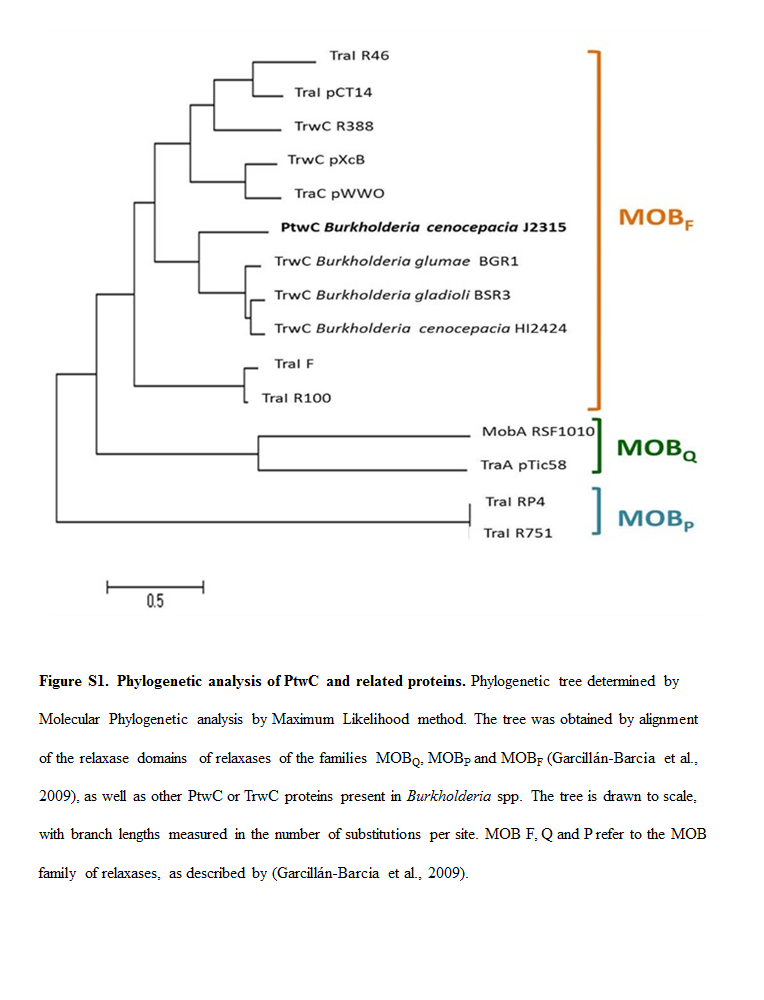

Supplement: Supplementary file 2 [file Image1.TIF]
